# Spatial Distributions and Intrinsic Influence Analysis of Cr, Ni, Cu, Zn, As, Cd and Pb in Sediments from the Wuliangsuhai Wetland, China

**DOI:** 10.3390/ijerph191710843

**Published:** 2022-08-31

**Authors:** Huilan Zhang, Piaopiao Liang, Ying Liu, Xinglei Wang, Yahong Bai, Yunxin Xing, Chunli Wei, Yuanyuan Li, Yiming Liu, Yu Hu

**Affiliations:** 1 College of Life and Environmental Sciences, Minzu University of China, Beijing 100081, China; 2 China National Environmental Monitoring Centre, Beijing 100012, China; 3 Beijing Engineering Research Center of Food Environment and Public Health, Minzu University of China, Beijing 100081, China

**Keywords:** spatial distributions, intrinsic influence analysis, potentially toxic elements, ecological risks

## Abstract

The spatial distributions of Cr, Ni, Cu, Zn, As, Cd and Pb (potentially toxic elements, PTEs) in sediments and intrinsic influence factors from the Wuliangsuhai wetland of the Hetao Irrigation District, China were studied in this work. The results showed that excluding Zn, the total contents of other PTEs were higher than the background values, of which As (39.26 mg·kg^−1^) and Cd (0.44 mg·kg^−1^) were six-fold and seven-fold higher, respectively. Especially, the high levels of Cd (70.17%), Pb (66.53%), and Zn (57.20%) in the non-residual fraction showed high bioavailability and mobility. It indicated that PTEs can enter the food chain more easily and produce much toxicity. Based on *I*_geo_, ICF, and MRI, the contamination of As was the most serious in the middle areas (MDP) of the wetland, and its risk was up to moderately strong. Cd and Pb posed moderate and considerate risk, respectively. Furthermore, 29.50% and 55.54% risk contribution ratio of As and Cd, respectively, showed that they were the dominant contaminants. In addition, the positive correlation between sand, OM, and total contents and chemical fractions of PTEs by using PCM, RDA, and DHCA indicated that physicochemical properties could significantly influence the spatial distributions of PTEs. The work was useful for assessing the level of pollution in the study area and acquiring information for future and possible monitoring and remediation activities.

## 1. Introduction

Potentially toxic elements (PTEs) contamination has become a widespread concern, especially in aquatic ecosystems [1,2]. PTEs pose a great threat to the aquatic environment due to their toxic effects and long-term accumulation potential in sediments and organisms [3,4,5]. In recent years, PTEs produced by human activities have been discharged into aquatic ecosystems by various paths including surface runoff and atmospheric dustfall, and more than 90% of the PTEs are ultimately immobilized in the sediments [6,7,8]. However, when the capacity of sediments to absorb PTEs exceeds its maximum load under certain environmental conditions, the sediments will desorb them into the overlying waters, resulting in serious hazard to health of aquatic ecosystems and humans [9,10].

In China, PTEs contamination in sediments has been extensively studied. In order to evaluate the pollution level of the environment [11], the total contents of PTEs are used as an environmental management standard [12,13]. The chemical fractions strongly influence the chemical activity, mobility, bioavailability, and potential toxicity of PTEs [14,15] and can be used to assess the ecological risks of PTEs in sediments. Therefore, it can provide more detailed and useful information about the amounts of metals available to organisms, mobility, and risk assessment [16,17]. Thus, a three-stage sequential extraction procedure proposed by the European Community Bureau of Reference (BCR) has been used to determine PTEs fractionations in soil and sediments, which were the acid-soluble (F1), reducible (F2), oxidizable (F3), and residual fractions (F4), respectively [18,19]. Among them, the former three parts are closely related to the bioavailability and mobility of PTEs, while the residual fraction can remain stable in sediments for a long time [20,21,22].

To evaluate the potential ecological risks of PTEs in sediments, the geo-accumulation index (*I*_geo_), enrichment factor (EF), and potential ecological risk index (PERI) have been widely applied [23,24]. Among them, *I*_geo_ can quantify the pollution degree of PTEs barely based on total PTEs contents; PERI merely takes toxicities and total PTEs contents into consideration to assess potential ecological risks [25]. Recently, a modified potential ecological risk index (MRI), which simultaneously considered the influences of toxicities, total contents, and bioavailability of PTEs, was proposed to better reflect environmental conditions [26,27]. In addition, individual contamination factor (ICF) is also an evaluation index taking non-residual fractions into consideration to accurately identify the potential bioavailability of PTEs [28]. Therefore, it would be interesting to apply above methods to comprehensively evaluate PTEs pollution from different perspectives.

The Wuliangsuhai wetland is the largest freshwater wetland in the Yellow River basin, located at the end of the plain of the Hetao Irrigation District, Inner Mongolia, China [29]; it has been a major recipient of irrigation runoff through the drainage channels including agricultural, industrial, and domestic wastewater without effective treatment [30]. Irrigation runoff accounts for 96% of the total volume of water in the wetland, leading to an increase in the degree and extent of PTEs pollution [31]. Meanwhile, because of the resuspension and migration of the PTEs, their contamination will directly affect the water security for the lower reaches of the Yellow River, which has weak self-purification ability in the low-water period [32]. In addition, previous studies have suggested that the sediments physicochemical properties, such as pH, iron oxide, and total organic carbon, can affect PTEs chemical fractions and behavior, which is important for evaluating the potential impact on the aquatic biota [33,34,35]. However, there were few studies to focus on the relationships between various physicochemical properties of sediments and chemical fractions of PTEs in the Wuliangsuhai wetland.

The aims of this study were to (1) investigate the ecological risks of Cr, Ni, Cu, Zn, As, Cd, and Pb in sediments with indices of the *I*_geo_, EF, ICF, and MRI; (2) quantify the chemical fractions and bioavailability of PTEs by BCR sequential extraction procedure; and (3) evaluate the influence of physicochemical properties of sediments on PTEs and their chemical fractions using multivariate statistical analysis. The results will provide reference data necessary for managing and protecting wetland ecosystem in the Hetao Irrigation District.

## 2. Materials and Methods

### 2.1. Study Area and Sample Collection

**Figure 1:**
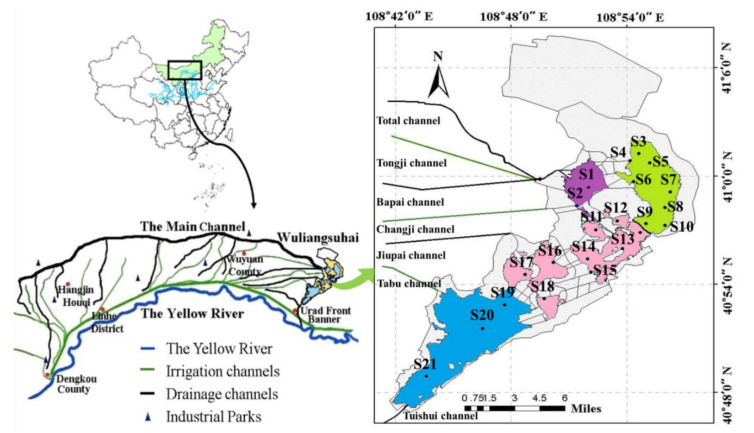
Location of the Wuliangsuhai wetland in Hetao Irrigation District, China. (The data sets of the Wuliangsuhai wetland and Hetao irrigation system are modified from Google Earth; the data set of Chinese Map is provided by Data Center for Resources and Environmental Sciences, Chinese Academy of Sciences (http://www.resdc.cn, accessed on 25 January 2019)).

The Wuliangsuhai wetland (108°43′~108°57′ E, 40°27′~40°03′ N) is located in the Bayannaoer Prefecture of the Inner Mongolia Autonomous Region in China at the eastern end of the Hetao irrigation system [36] (Figure 1). The total area is approximately 333.48 km^2^, 119 km^2^ of which is covered with *P. australis*, and there is 290 km^2^ of open water [37,38,39] due to the division of it. The average depth is only about 1m and the maximum depth is less than 4 m. In addition, it lies in a desertified and semi-desertified area with a mid-temperate continental arid and semi-arid monsoon climate. The average annual rainfall is 224 mm, and evaporation is 1502 mm. The frost-free period lasts 152 days and the ice-locked period lasts for about 5 months [40]. The sediment in the northern part of the Wuliangsuhai wetland is basically black or brown and smells bad, while in the southern part, sediment is gray and black. The moisture content of the fresh sediment ranges from 46.17% to 60.63% [41,42].

The Wuliangsuhai wetland is an important part of the agricultural irrigation and drainage system, the residuals of chemical fertilizers and waste materials of mineral processing enterprises near drainage channels discharge into the Wuliangsuhai wetland, leading to PTEs pollution for the wetland ecosystem and flowing into the down-stream areas of the Yellow River through the outlet of the wetland [43]. There are four areas in the Wuliangsuhai wetland. Xidatan (XDT) is located in the northwest of the wetland, and is most affected by external pollution which enters directly by the irrigation and drainage channels; the northeastern areas (NEA) are the nature reserve, which is minimally affected by human activities; in the middle part (MDP) of the wetland, there are six open-water zones by the opening of artificial grid channels; and the fish culture area (FCA) is located in the only outlet of the wetland.

Based on this, the sampling sites in this study are also divided into above four regions (Figure 1), there were two sampling sites in XDT (S1, S2), eight in NEA (S3–S10) and MDP (S11–S18), respectively, and three in FCA (S19–S21). The 21 sediments samples were naturally dried, ground, and sieved through a 200-mesh nylon sieve to prepare for the determination of sediments physicochemical prosperities, Cr, Ni, Cu, Zn, As, Cd, and Pb contents and chemical fractions.

### 2.2. PTEs Contents and Chemical Fractionations

Total PTEs contents were detected by ICP-MS after digesting sediments samples with HNO_3_-HF-H_2_O_2_ solution [44]. The certified reference materials (sediment: GBW07307(GSD-7a)) was used for quality control, and the recovery ranged from 90.1% to 112.4%.

Chemical fractions of Cr, Ni, Cu, Zn, As, Cd, and Pb in the sediments were determined using the modified BCR sequential extraction procedure according to a previous study [45,46] (Table S1). The sum of total extracted quantities of Certified Reference (CRM) heavy metals was obtained by four steps (Table S1), and the direct pseudo-total quantity of the sediment-based Certified Reference Material BCR 701 by microwave digestion with a mixture of HNO_3_-HF-H_2_O_2_ to check the accuracy of the fractionation procedure by the following equation:

(1) Recovery=(F1+F2+F3+F4)/pseudo−total content ×100%

### 2.3. Eco-Risk Assessment

#### 2.3.1. Geo-Accumulation Index (*I*_geo_) and Enrichment Factor (EF)

The index *I*_geo_ and EF have been used to gauge the contamination levels of PTEs [47,48,49]. The *I*_geo_ is calculated using the following equation:

*I*_geo_ = log_2_[*C*_n_/(1.5*B*_n_)] (2)

where *C*_n_ is the content of metal (*n*) in sediments and *B*_n_ is the geochemical background content of metal (*n*). The sediments quality was categorized in seven classes shown in Table S3.

The calculation formula of the index EF is given as follows:

(3) $$\mathrm{EF}={\left(\mathrm{Metal}\;/\;\mathrm{Al}\right)}_{\mathrm{sample}}/{\left(\mathrm{Metal}\;/\;\mathrm{Al}\right)}_{\mathrm{background}}$$

where (Metal/Al)_sample_ is the metal to Al content ratio in the PTEs and where (Metal/Al)_background_ is the geochemical background content of PTEs to Al ratio. The enrichment degree of PTEs in sediments could be graded into seven categories (Table S3).

#### 2.3.2. Individual Contamination Factor (ICF) and Global Contamination Factor (GCF)

The index ICF is used to assess the contamination of single element as an important index to evaluate the potential ecological risk of PTEs [28,50], and the value of ICF is calculated by the following equation:

ICF = (F1 + F2 + F3)/F4(4)

(5) $$\mathrm{GCF}=\sum_{i=1}^{n}{\mathrm{ICF}}_{i}$$

The GCF reflects the overall potential risk posed by the complex and adverse influence of PTEs to the environment. The categories of ICF and GCF are listed in Table S4.

#### 2.3.3. The Modified Potential Ecological Risk Index (MRI)

A modified potential ecological risk index (MRI) was used to assess the potential ecological risk of PTEs [26,51]. The calculation of MRI is shown as follows:

(6) $${\mathrm{MRI}}_{i}=\sum_{i=1}^{n}{\mathrm{MEI}}_{i}{=T}_{i}\times {\tilde{C}}_{d}^{i}\times \beta /{\tilde{C}}_{r}^{i}$$

(7) β=Aα+(1 − A)

where MRI, MEI, ${\tilde{C}}_{d}^{i}\;$*,* and ${\tilde{C}}_{r}^{i}\;$ are the modified forms of PERI, EI, $C_{d\;}^{i},$ $C_{r\;}^{i}$ and, respectively; β  is the modified index of HM contents; A is the percentage of F1 fraction in the sum of four fractions (F1 + F2 + F3 + F4); and *α* is the toxic index corresponding to different ratios of F1 fraction [20]. Similar to the classification of PERI, the categories of MRI_i_ and MEI_i_ are listed in Table S5.

### 2.4. Multivariate Statistical Analysis

In this study, PTE contents are evaluated by implementing multivariate statistical analyses including the correlation matrix (PCM), redundancy analysis (RDA), and dual hierarchical clustering analysis (DHCA) [52,53]. PCM is conducted to identify the degree of potential correlations between the variables, including total contents, chemical fractions of PTEs, and the physicochemical properties of sediments using SPSS 19.0 [33]. RDA is used to test for correlations between the selected sediments physicochemical properties and PTEs fractions using the Canoco 5. DHCA is conducted by Heml, which is a toolkit for illustrating heat maps [54], and all of the variables conducted in DHCA are firstly normalized through transformation.

## 3. Results and Discussion

### 3.1. Physicochemical Properties of Sediments

Physicochemical properties of sediments in four sampling areas were listed in Table S6. The pH is a key parameter controlling heavy metals transfer behavior in sediments [55]; it ranged from 7.86 to 8.70 with an average of 8.12. According to maximum retention of cationic metals occurs at pH > 7, the PTEs with cationic behavior had higher portion in the Wuliangsuhai wetland [56]. The sediments were mainly composed of silt, but percentages of sand in the NEA (11.71%) and MDP (10.58%) were higher than that in the FCA (2.14%), which had more clay (15.50%). The percentages of OM, Fe, and Mn showed a decreasing trend from XDT to FCA. The sediments organic matter (SOM, 8.91–16.74%) generally acted as a major sink for PTEs because of its strong complexing capacity [44].

### 3.2. PTEs Contents in Sediments

**Figure 2:**
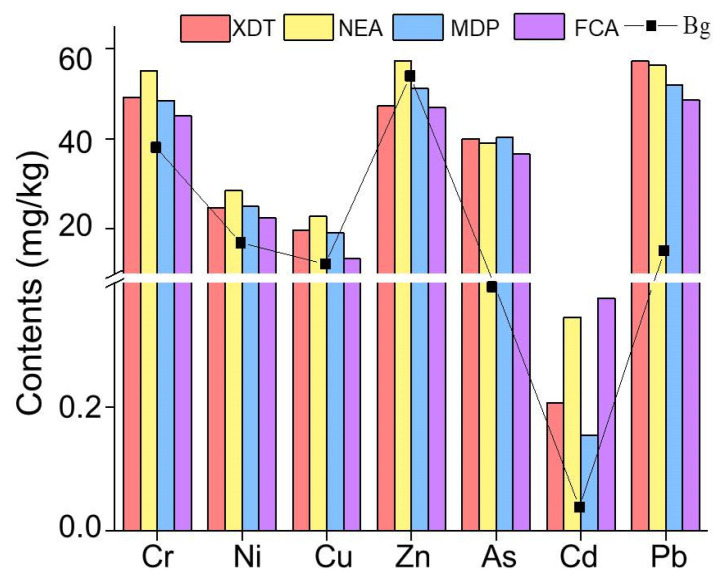
The contents of PTEs in sediments of the Wuliangsuhai wetland.

In the 21 sampling sites, PTEs contents followed the sequence: Pb > Zn > Cr > As > Ni > Cu > Cd (Figure 2) with average values of 53.62, 52.51, 50.60, 39.26, 25.95, 19.80, 0.26, mg·kg^−1^, respectively. The results showed that As, Cd, Pb, Cr, Cu, and Ni were higher than the background values, especially for As and Cd, which were six-fold and seven-fold higher, respectively. However, the average contents of Zn, except at the NEA, were lower than the background values. Except Cd, Zn, and Pb, the contents of other PTEs in the XDT were almost same as in MDP, while the average contents of Cr, Ni, Cu, Zn, and Cd in the NEA were higher than those in other three areas. A comparison of the PTEs in this work with those by other studies in the Wuliangsuhai wetland (Table S7) indicated that the content levels of Cd, Pb, and As were higher than that of metals [57,58]. That is likely a result of alteration of hydrological processes influenced by human activities in the irrigation region, rapid development of the smelting activity, and heavy inputs of agrochemicals and fertilizers from upstream agricultural drainage.

### 3.3. Chemical Fractions of PTEs in Sediments

**Figure 3:**
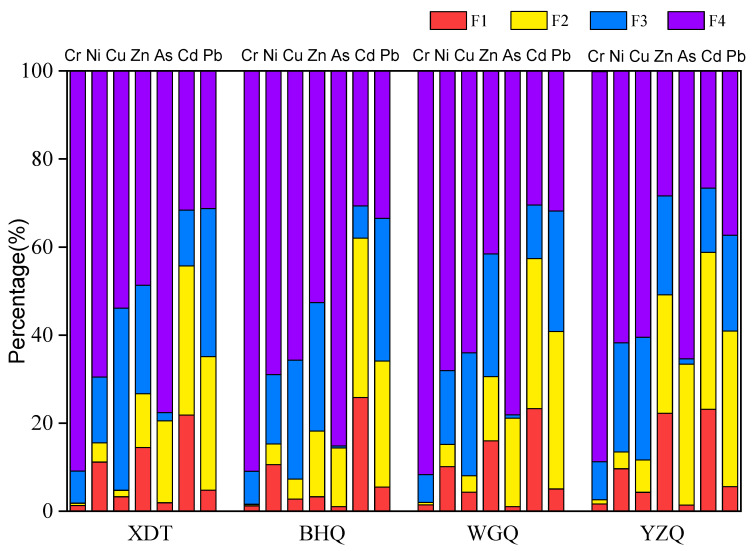
Chemical fractions of PTEs in sediments of the Wuliangsuhai wetland.

In this study, a modified BCR sequential extraction procedure (Figure 3) was used to obtain the chemical fractions of PTEs, which are more essential parameters in the sediments than the total contents in terms of their bioavailability, potential sources, and environmental behaviors [59,60]. As shown in Table S2, the overall sum of four fractions was in good agreement with direct pseudo-total contents, and the recoveries ranged from 92.3% to 123.0%, indicating the modified BCR method was reliable.

The results exhibited that distributions of F1, F2, F3, and F4 chemical fractions ranged from 0.95–65.56%, 1.41–48.44%, 3.04–38.96%, and 13.75–94.66%, respectively (Table S8). The non-residual of Cd was the highest, but Cr was the lowest. Moreover, the non-residual distributions of Zn and As in four sampling areas were similar, followed by FCA > MDP > XDT > NEA. This result showed that the pollution of the two PTEs mainly originated from human activities and the management of protected areas was effective.

The F1 fraction reflected the exchangeable and bioavailable properties of PTEs. In this study, the F1 fraction of Cd ranged from 15.67% to 30.77%, indicating that Cd had higher mobility and bioavailability than that of other PTEs. The ionic radius of Cd (0.97 Å) was similar to Ca (0.99 Å) [61,62], which caused the Cd to co-precipitate with carbonate minerals [62]. The F2 fraction was associated with metals that combined with Fe and Mn oxides and might be released under the reducing conditions. In this study, Pb had the highest level of the F2 fraction (48.33%), followed by Zn (44.42%) and Cd (40.22%), respectively. Cu primarily existed in the F3 fraction, ranging from 15.12% to 44.45%, which demonstrated that tendency of Cu bound to organic matter in sediments was relatively high [27]. Previous studies had showed that Cu can easily form complexes with organic matter given the high stability constant of organic-copper compounds [63,64]. In addition, the high percentages of the F3 fraction (14–44.96%) for Pb and Zn showed that there were large amounts of metals being associated with humus reflecting the organic matter-rich pollution caused by biological activities and human emissions [65]. The monitoring of PTEs should be given special care when the bottom sediments would be exposed to oxygen during the waterway dredging project; because the F3 fraction was high, PTEs can be released from sediments under oxidizing conditions, such as environmental dredging or water quality recovery. On the contrary, Cr and As were essentially found in F4 fraction with the high proportion of 94.66% and 89.13%, respectively. It indicated that Cr was strongly bound in mineral lattices in the sediments. In other words, Cr posed little potentially hazardous risk to aquatic organisms and environment, because it was difficult to release under natural conditions.

### 3.4. Risk Assessments for the Sediments

#### 3.4.1. Geo-Accumulation Index (*I*_geo_) and Enrichment Factor (EF)

**Figure 4:**
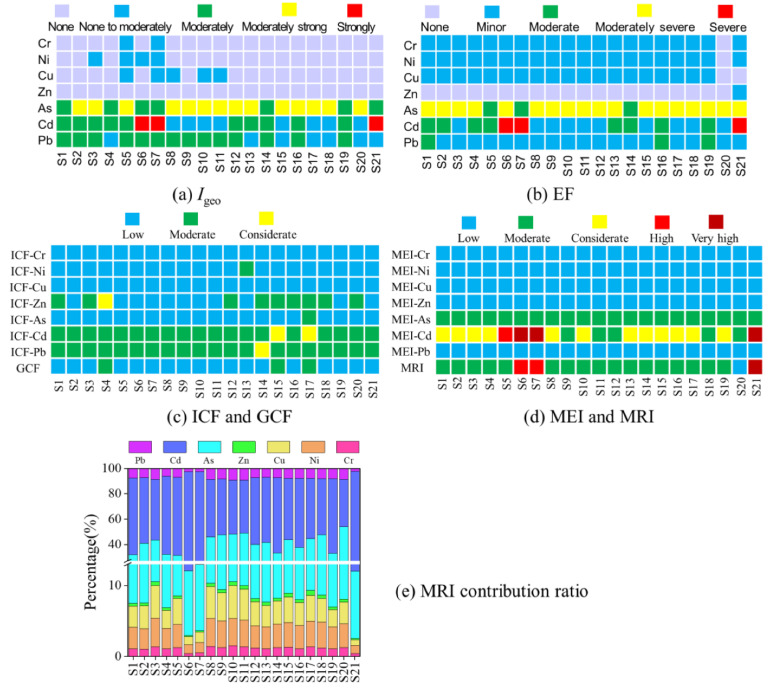
Contamination assessments of PTEs in sediments of the Wuliangsuhai wetland by *I*_geo_, EF, ICF and MRI.

In Figure 4a, the results of *I*_geo_ ranged from “none” (uncontaminated) to “Strongly”; the average *I*_geo_ values of PTEs followed the order of As (2.09) > Cd (1.43) > Pb (1.08) > Cu (−0.11) > Ni (−0.12) > Cr (−0.25) > Zn (−0.71). The dominating contamination was As with “moderately strongly” degree, especially in the MDP. Cd in S6, S7, S21 was up to the level of “strongly contaminated”, while Cr, Ni, and Zn were the relatively least contaminated with “none to moderately”.

The enrichment factor (EF) for PTEs followed the order of As (5.82) > Cd (4.75) > Pb (2.87) > Cu (1.27) > Ni (1.25) > Cr (1.14) > Zn (0.83), respectively, which was consistent with *I*_geo_. It indicated that anthropogenic activities contributed greatly to the high enrichment of As, Cd, and Pb (EF > 1.5, Figure 4b). The EF of As was categorized as “moderately severe” in all sampling sites. For Cd, severe pollution level appeared at S6, S7, S21.

#### 3.4.2. Individual Contamination Factor (ICF) and Global Contamination Factor (GCF)

ICF is an evaluation index only considering the proportion of non-resident, and can reflect bioavailability of PTEs and the level of harm to aquatic organisms. Figure 4c showed the ICF of each heavy metal and GCF, where ICF values of Cr, Ni, Cu, and As were <1, which meant low risk to the aquatic ecosystem, but Ni at S13 and As at S17 were moderate. Due to a large proportion of Cr, Ni, Cu, and As in the F4, they had low mobility. In addition, the risk of Zn was moderate at most sites because of its low ICF (except S4 and S17-considerate), and Cd and Pb also had moderate risk to the environment (except Cd at S15 and S17, Pb at S14-considerate).

The GCF was the sum of ICF for Cr, Ni, Cu, Zn, As, Cd, and Pb, the results showed moderate contamination at S4, S15, and S17 (6 < GCF < 12); even Zn had lower total contents than background values. Since GCF is a speciation index to reflect the general environmental risks of a specific site [56], it is of great significance to pay more attention to the two indexes in order to accurately evaluate PTEs mobility and bioavailability.

#### 3.4.3. The Modified Potential Ecological Risk Index (MRI)

As a newly modified potential ecological risk index, MRI simultaneously considered the levels, bioavailability, and toxicities of heavy metals in sediments. The MEI values (Figure 4d–e) of PTEs followed the order of Cd (166.01) > As (64.15) > Pb (15.92) > Cu (7.11) > Ni (7.06) > Cr (2.54) > Zn (0.96), respectively, and corresponding risk contribution ratios were 55.54%, 29.49%, 7.14%, 3.18%, 3.11%, 1.12%, and 0.42%, respectively. Since F1 was easily transformed and migrated with the environmental changes, it could be absorbed directly by the biotas; thus, the Cd in this fraction might contribute significantly to the biota the PTEs posed, which were consistent with the bioavailability of PTEs in sediments due to the highest MRI value. From the risk contribution ratios, As and Cd were the dominant contaminants, which were consistent with the results of *I*_geo_.

Therefore, it was not possible to comprehensively assess the actual ecological risk of the PTEs by a single assessment method, so the multiple assessment indexes, simultaneously taking the levels, toxicities, and bioavailability of PTEs into consideration, were useful for improvement of ecological risk assessment and management of heavy metals in sediments [66].

### 3.5. Multivariate Statistical Analysis

#### 3.5.1. Pearson Correlation Matrix (PCM)

PCM can explore potential correlations between PTEs and the physicochemical properties of sediments. The results showed that total contents and chemical fractions of PTEs had different degrees of correlation with the physicochemical properties (Table 1).

As shown in Table 1, firstly, there were strongly positive correlations between the pH and the F4 fraction of Cr, Cu, and total contents of Ni, Cu, and Zn (*p* < 0.01), suggesting the change of pH would cause increase of resident fraction of Cr and Cu. This meant that both Cr and Cu were stable and had low mobility due to the decrease of bioavailability fraction. Secondly, a negative correlation was found between clay and Cr, Ni, Cu, Zn, and As (*p* < 0.01), but sand was positively related to Cr, Ni, Cu, and Zn; it was consistent with the lower contents of Cr, Ni, Cu, Zn, and As in FCA area with little percentage of sand. Thirdly, organic matter (OM) showed a positive correlation with the F3 fractions of Cu and Pb, whereas the F1 of Cr showed a negative correlation with OM (*p* > 0.05). Thus, the OM significantly influenced the chemical fractions of PTEs in sediments. Moreover, the other fractions of PTEs may transfer to F3 fraction with higher contents of OM in sediments, leading to the reduction of ecotoxicity and transfer capability [67], but the toxicity of Cr could increase; this is due to high specific surface of sand and organic matter as well as loaded surfaces and high cation exchange capacity of these grains [68]. Lastly, the contents of Fe and Mn were not strongly correlated with the fractions of PTEs in sediments in this study [69]; we inferred that the sediments with a high proportion of nutrient elements might change the redox potentials of the sediments and caused the unstable reducible fraction to be transferred to other fractions [70,71].

The correlation indicated that pH, particle size, and OM might play a major role in the migration and transition of PTEs. The strongly positive correlations among these parameters and PTEs indicated that these pollutants had the mutual dependence with physicochemical properties of sediments and the same transport behavior [11].

#### 3.5.2. Redundancy Analysis (RDA)

**Figure 5:**
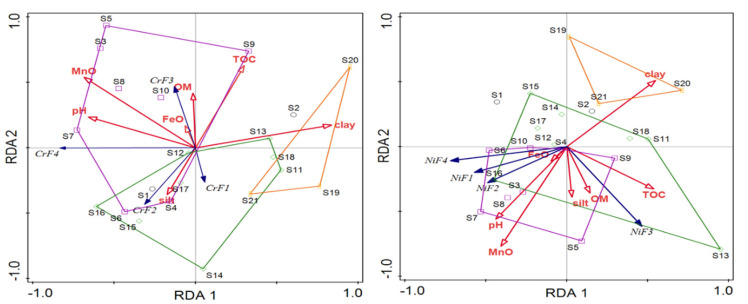

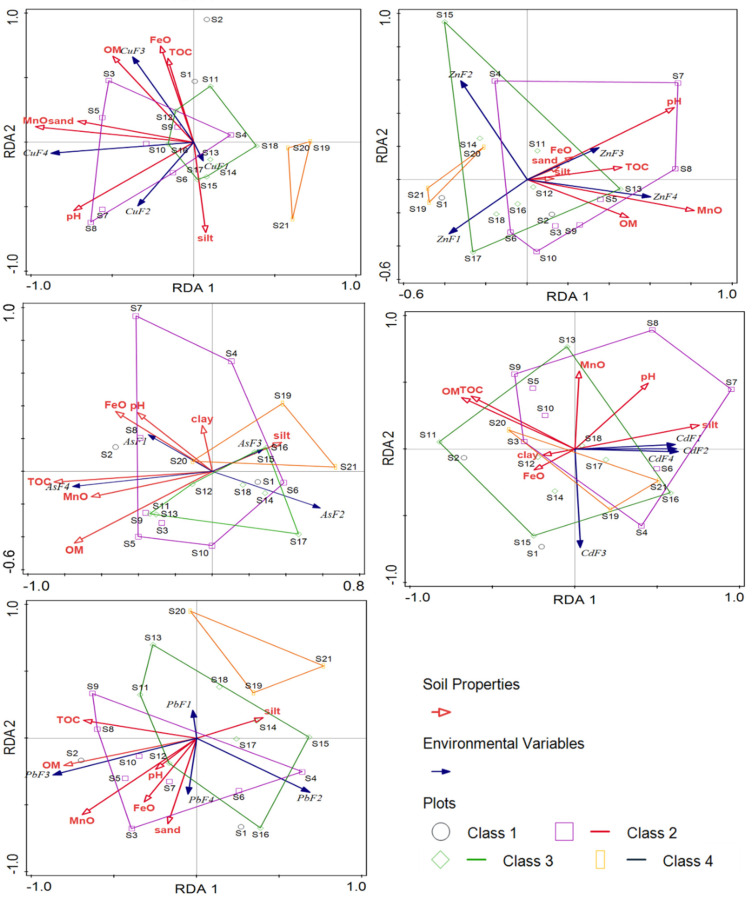
RDA of fractional contents of PTEs associated with sediments physicochemical properties of the Wuliangsuhai wetland (Class 1—XDT; Class 2—NEA; Class 3—MDP; Class 4—FCA).

RDA can further explore the relationship between the chemical fractions of PTEs and the physicochemical properties of the sediments. In Figure 5, the RDA showed that different physicochemical properties of the sediments had different degrees of influence on the fractions of PTEs. In the NEA and MDP areas, the OM showed significant positive correlations with the F3 fractions of Ni, Cr, Cu, and Pb. This result further affirmed that the F3 fractions were mainly associated with anthropogenic contributions, and the contents of organics played a key role in determining the fractions of PTEs [72,73].

Additionally, the F1 and F2 fractions of Cr and Cd were heavily affected by the silt in sediments, suggesting that fine-sized sediments were bound to non-resident fractions. The small particles represented higher surface area to volume ratios for better retention of the fractions, leading to higher toxic to aquatic organisms [74].

#### 3.5.3. Two-Way Hierarchical Cluster Analysis (DHCA)

**Figure 6:**
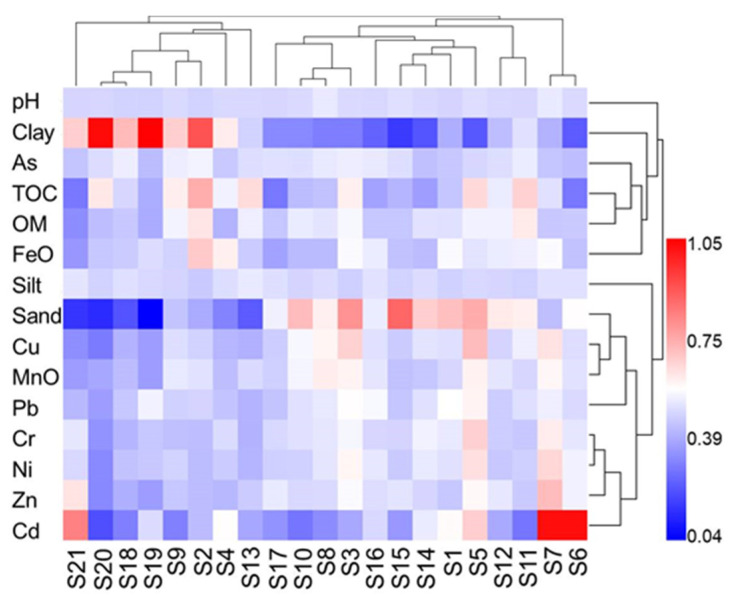
Two-way hierarchical cluster analysis for the heavy metals, sampling sites, and properties of sediments.

DHCA can identify the degree of influence between the total contents of PTEs and the physicochemical properties. In Figure 6, the horizontal dendrogram was divided into three clusters: (1) pH; (2) clay, As, TOC, OM, and FeO; (3) silt, sand, MnO, Cu, Pb, Zn, Cr, Cd, and Ni. The cluster 2 indicated that clay, OM, FeO, and TOC were significantly related to the As concentrations, which tended to associate with fine particles and organic matter, especially organic carbon in the sediments. The cluster 3 showed that silt, sand, and MnO were also essentially related to the Cu and Pb concentrations, which demonstrated that Cu and Pb might have similar geochemical behaviors. The sampling sites in the vertical dendrogram were in three groups. Group 1 included all sites at the FCA and S18 in MDP, that is S19, S20, S21, and S18, group 3 was composed by S6 and S7, whereas the group 2 contained the rest sites. The first group showed high levels of PTEs pollution due to the similarity of particle composition (similar color pattern in heat map). It can be seen from the heat map that the higher percentage of sand, FeO, and MnO will result in the more serious pollution of PTEs, which was in agreement with the result of PCM.

## 4. Conclusions

The results of this study showed that the total contents of As and Cd were six-fold and seven-fold higher than the background values, respectively, and they were higher than these of previous studies, indicating PTEs pollution was increasing in the area. High levels of non-residual fraction of Cd (70.17%), Pb (66.53%), and Zn (57.20%) showed high availability and mobility, while high levels residual fraction of As (76.65%) and Cr (90.50%) mainly existed in the residual fraction with low availability; relatively high levels of oxidizable fraction of Cu (31.02%) and Pb (32.79%) suggested that these PTEs should receive attention during the dredging project of the Wuliangsuhai area. Multiple assessment methods illustrated that the sediments were contaminated by As, Cd, Zn, Ni, and Pb. Among them, Cd contamination was the most serious in BHQ, and the pollution level of As was the highest in WGQ. Furthermore, 29.50% and 55.54% risk contribution ratio of As and Cd indicated that they were the dominant contaminants (moderately–moderately strong and considerate–very high) in the study areas, respectively. Pollution sources of As and Cd were mainly related to the waste discharge from local sulfuric acid plants and smelters. In addition, As came from agricultural non-point source pollution in the Hetao irrigation area, too. It was also suggested that in order to evaluate the ecological risks of PTEs in wetland sediments more effectively, the total contents and chemical fractions should be taken into consideration. The strong correlation between physicochemical properties (especially for pH, grain size, and OM) and total contents, chemical fractions of PTEs in the multivariate statistical analysis demonstrated that physicochemical properties can affect the distributions and behaviors of PTEs; especially, a positive correlation among sand and OM with the most PTEs indicated the two physicochemical properties played an important role in determining the contamination and chemical fractions of heavy metals. Thus, the physicochemical properties of sediment in Wuliangsuhai should be monitored periodically, especially focusing on OM and pH. In order to control the pollution more effectively, the absorption of PTEs in non-residual state from the sediment by plants still needs more in-depth investigation.

## Figures and Tables

**Table 1:** Pearson correlation analysis among sediments physicochemical properties and total contents, chemical fractions of PTEs.

PTEs		pH	Clay	Silt	Sand	TOC	OM	Fe	Mn
Cr	CrTotal	0.522 *	−0.633 **	0.120	0.538 *	−0.199	0.015	0.055	0.554 **
	CrF1	−0.110	0.230	0.574 **	−0.551 **	−0.478 *	−0.777 **	−0.309	−0.442 *
	CrF2	−0.073	−0.270	0.160	0.166	−0.398	−0.437 *	−0.063	−0.246
	CrF3	0.189	−0.022	−0.143	0.104	0.253	0.185	0.086	0.332
	CrF4	0.550 **	−0.699 **	0.146	0.585 **	−0.251	0.012	0.052	0.570 **
Ni	NiTotal	0.565 **	−0.613 **	0.151	0.501 *	−0.150	0.082	0.138	0.646 **
	NiF1	0.341	−0.313	0.110	0.236	−0.194	−0.137	0.224	0.324
	NiF2	0.407	−0.583 **	−0.154	0.648 **	−0.040	0.121	0.140	0.454 *
	NiF3	0.102	−0.079	0.264	−0.077	0.464 *	0.337	0.063	0.246
	NiF4	0.402	−0.428	0.044	0.384	−0.394	−0.098	0.017	0.407
Cu	CuTotal	0.586 **	−0.659 **	−0.188	0.740 **	0.193	0.545 *	0.275	0.901 **
	CuF1	−0.029	−0.378	0.264	0.210	−0.525 *	−0.317	−0.073	−0.144
	CuF2	0.504 *	−0.463 *	0.186	0.337	−0.315	−0.186	−0.332	0.218
	CuF3	−0.069	−0.071	−0.488 *	0.349	0.511 *	0.611 **	0.613 **	0.439 *
	CuF4	0.694 **	−0.676 **	−0.014	0.656 **	0.052	0.396	0.057	0.858 **
Zn	ZnTotal	0.580 **	−0.604 **	0.325	0.392	−0.265	−0.073	−0.116	0.491 *
	ZnF1	−0.305	−0.068	0.044	0.039	−0.369	−0.198	−0.223	−0.167
	ZnF2	0.147	−0.138	0.151	0.045	−0.249	−0.525 *	−0.125	−0.305
	ZnF3	0.443 *	−0.366	0.026	0.336	−0.056	0.251	0.080	0.360
	ZnF4	0.419	−0.177	0.156	0.080	0.246	0.225	0.101	0.518 *
As	AsTotal	−0.015	0.010	−0.365	0.200	0.531 *	0.596 **	0.199	0.363
	AsF1	−0.222	0.578 **	−0.474 *	−0.281	0.573 **	0.193	0.455 *	−0.247
	AsF2	−0.31	−0.081	0.180	−0.026	−0.498 *	−0.360	−0.406	−0.340
	AsF3	−0.419	0.313	−0.234	−0.165	−0.104	−0.127	0.220	−0.323
	AsF4	0.267	0.011	−0.305	0.165	0.663 **	0.621 **	0.376	0.506 *
Cd	CdTotal	0.287	−0.225	0.432	−0.033	−0.361	−0.361	0.052	0.105
	CdF1	0.310	−0.164	0.447 *	−0.100	−0.386	−0.361	−0.132	0.076
	CdF2	0.280	−0.114	0.464 *	−0.158	−0.372	−0.426	−0.135	0.016
	CdF3	−0.317	−0.025	−0.133	0.101	−0.332	−0.258	0.124	−0.374
	CdF4	0.251	−0.096	0.484 *	−0.187	−0.398	−0.463 *	−0.16	−0.024
Pb	PbTotal	0.244	−0.409	−0.207	0.512 *	−0.014	0.3	0.36	0.581 **
	PbF1	−0.002	0.226	−0.176	−0.115	−0.053	−0.049	−0.021	−0.207
	PbF2	−0.032	−0.273	0.174	0.161	−0.561 **	−0.497 *	−0.109	−0.194
	PbF3	0.228	−0.187	−0.446 *	0.437 *	0.596 **	0.873 **	0.457 *	0.715 **
	PbF4	0.155	−0.111	0.162	0.013	−0.151	−0.122	0.107	0.316

*. Correlation is significant at the 0.05 level. **. Correlation is significant at the 0.01 level. Bold means high correlation.

## Data Availability

Not applicable.

## Supplementary Materials

The following supporting information can be downloaded at: https://www.mdpi.com/article/10.3390/ijerph191710843/s1, Table S1: The BCR sequential extraction used for operational speciation of HMs; Table S2: Recoveries (%) of reference material BCR-701 after four extracted contents (mean ± SD, *n* = 3, mg/kg, dry weight); Table S3: Contamination categories based on enrichment factor (EF) and geo-accumulation index (*I*_geo_); Table S4: Indices and grades of the individual contamination factor (ICF) and GCF; Table S5: Indices and grades of potential ecological metals contamination; Table S6: Physicochemical characteristics and total HMs contents of the sediments in four areas from the Wuliangsuhai wetland; Table S7: HMs concentrations in sediments of the Wuliangsu wetland in recent years (mg/kg); Table S8: The chemical fraction contents of HMs in the sediments from the Wuliangsuhai wetland.
