# Supplementary material for: Spatial Distributions and Intrinsic Influence Analysis of Cr, Ni, Cu, Zn, As, Cd and Pb in Sediments from the Wuliangsuhai Wetland, China
Source: Int J Environ Res Public Health. 2022 Aug 31;19(17):10843. doi: 10.3390/ijerph191710843 (PMC9518466; doi:10.3390/ijerph191710843)
Supplement: Supplementary file 1 [file ijerph-19-10843-s001.zip › ijerph-1864703-supplementary.pdf]

Supplementary Material

Table S1

The BCR sequential extraction used for operational speciation of HMs

Step	Chemical fractions	Extractant	Conditions
F1	Acid-soluble	0.11 M CH_3COOH	Shake 16 h at RT
F2	Reducible	0.5 M $NH_2OH·HCl$ at pH 2.0	Shake 16 h at RT
F3	Oxidizable	30% H_2O_2	Shake 1 h at RT and 1 h at 85 °C
		1.0 M NH_4OAc at pH 2.0	16 h at RT
F4	Residual	a mixture of HNO_3-HF-H_2O_2	Microwave digestion

RT: room temperature.

Table S2

Recoveries (%) of reference material BCR-701 after four extracted contents (mean ± SD, *n*=3, mg/kg, dry weight)

Metals	Sum of four extracted contents (F1+F2+F3+F4)	Pseudo-total contents	Recovery (%)
Cr	290.94 ± 3.94	315.20 ± 15.72	92.3
Ni	103.5 ± 0.8	108.7± 4.3	95.2
Cu	301.9 ± 4.8	254.0± 11.9	118.9
Zn	588± 2	478± 11	123.0
Cd	11.9± 0.2	12.8± 0.5	92.9
Pb	159.3 ± 1.3	156.0± 6.1	102.1

Table S3

Contamination categories based on enrichment factor (EF) and geo-accumulation index (I_{geo})

Enrichment factor			Geo-accumulation index		
Level	Value	Categories	Level	Value	Classification
I	EF < 1	None enrichment	0	$I_{geo} < 0$	None contaminated
II	1< EF <3	Minor enrichment	1	$0 < I_{geo} < 1$	None to moderately contaminated
III	3 < EF < 5	Moderate enrichment	2	$1 < I_{geo} < 2$	Moderately contaminated
IV	5 < EF < 10	Moderately severe enrichment	3	$2 < I_{geo} < 3$	Moderately to strongly contaminated
V	10 < EF < 25	Severe enrichment	4	$3 < I_{geo} < 4$	Strongly contaminated
VI	25 < EF < 50	Very severe enrichment	5	$4 < I_{geo} < 5$	Strongly to extremely contaminated
VII	EF > 50	Extremely severe enrichment	6	$I_{geo} > 5$	Extremely contaminated

Table S4

Indices and grades of the individual contamination factor (ICF) and GCF

ICF value	Grade of ecological risk of single metal	GCF value
ICF < 1	Low risk	GCF < 6
1 ≤ ICF < 3	Moderate risk	6 ≤ GCF < 12
3 ≤ ICF < 6	Considerate risk	12 ≤ GCF < 24
ICF ≥ 6	High risk	GCF ≥ 24

Table S5

Indices and grades of potential ecological metals contamination

E_r^i value	Grade of ecological risk of single metal	RI value	Grade of ecological risk of environment
E_r^i < 40	Low risk	RI < 150	Low risk
40 ≤ E_r^i < 80	Moderate risk	150 ≤ RI < 300	Moderate risk
80 ≤ E_r^i < 160	Considerate risk	300 ≤ RI < 600	Considerate risk
160 ≤ E_r^i < 320	High risk	RI > 600	Very high risk
E_r^i ≥ 320	Very high risk		

Table S6 Physicochemical characteristics and total HMs contents of the sediments in four areas from the Wuliangsuhai wetland

Sampling areas and background values		Physicochemical characteristics (%)								Total HM contents (mg/kg)						
		pH	Clay	Silt	Sand	TOC	SOM	Fe	Mn	Cr	Ni	Cu	Zn	As	Cd	Pb
XDT	S1	7.93	6.57	79.37	14.06	1.12	13.55	4.50	0.09	53.69	26.84	20.26	48.45	36.49	0.24	62.03
	S2	7.90	15.12	77.00	7.88	1.84	16.74	4.50	0.10	44.65	22.67	19.24	46.06	43.49	0.18	52.42
	**Mean**	**7.92**	**10.85**	**78.19**	**10.97**	**1.48**	**15.15**	**4.50**	**0.09**	**49.17**	**24.75**	**19.75**	**47.26**	**39.99**	**0.21**	**57.22**
NEA	S3	8.14	4.90	79.15	15.95	1.50	14.83	5.98	0.11	56.69	31.07	26.70	59.75	42.58	0.16	62.56
	S4	8.11	9.93	83.82	6.25	1.35	11.08	4.18	0.08	51.02	24.43	16.83	45.10	36.54	0.23	48.60
	S5	8.25	3.65	81.49	14.86	1.61	14.57	6.34	0.11	68.64	33.40	28.52	62.50	38.75	0.27	64.50
	S6	8.13	3.74	84.78	11.48	0.72	12.38	4.57	0.10	53.44	28.67	20.10	57.82	34.69	0.44	53.84
	S7	8.70	6.71	84.54	8.75	1.26	12.27	5.83	0.11	62.82	34.46	25.39	75.64	35.90	0.44	58.38
	S8	8.65	4.92	83.04	12.04	1.10	13.78	5.70	0.11	53.23	27.22	23.79	52.81	41.70	0.14	57.01
	S9	8.14	10.94	80.14	8.92	1.51	14.63	4.80	0.10	45.26	25.26	20.11	48.21	42.51	0.13	51.75
	S10	8.16	5.33	80.46	14.21	1.07	14.17	4.67	0.10	52.08	25.05	22.28	52.31	40.16	0.12	55.46
	**Mean**	**8.26**	**6.15**	**82.14**	**11.71**	**1.31**	**13.74**	**5.26**	**0.10**	**55.11**	**28.53**	**22.77**	**57.30**	**39.06**	**0.34**	**56.32**
MDP	S11	8.01	8.25	79.67	12.08	1.65	16.44	4.66	0.09	47.06	24.91	21.53	49.29	42.29	0.12	54.89
	S12	8.10	7.07	80.66	12.27	1.32	14.58	5.34	0.10	47.99	24.07	19.34	55.51	40.03	0.16	50.79
	S13	8.08	7.78	87.61	4.61	1.60	14.31	4.33	0.09	42.26	21.91	16.39	49.36	39.91	0.16	45.05
	S14	8.11	3.55	82.96	13.49	0.92	13.66	4.85	0.08	55.80	27.80	20.74	51.61	35.07	0.22	55.42
	S15	8.32	2.66	79.62	17.72	1.04	12.15	4.65	0.08	49.37	24.34	18.63	54.79	40.08	0.15	49.02
	S16	8.10	4.04	85.35	10.61	0.95	12.17	4.98	0.10	49.96	27.46	20.38	53.36	41.62	0.20	60.63
	S17	8.02	5.34	83.85	10.81	0.72	12.12	4.63	0.09	50.45	24.92	18.80	56.36	40.37	0.14	48.61
	S18	7.92	11.64	84.07	4.29	1.21	12.23	4.10	0.08	42.75	23.36	16.55	43.12	42.52	0.13	49.40
	**Mean**	**8.11**	**6.41**	**83.01**	**10.58**	**1.13**	**13.18**	**4.69**	**0.09**	**48.49**	**25.01**	**19.23**	**51.14**	**40.28**	**0.15**	**51.92**

	S19	7.86	17.93	81.28	0.79	1.00	10.71	3.60	0.07	46.54	24.13	14.75	39.23	34.29	0.20	59.36
FCA	S20	8.00	17.63	79.76	2.61	1.54	11.69	3.28	0.07	35.58	17.25	11.90	34.45	39.73	0.08	40.06
	S21	7.97	10.95	86.02	3.03	0.72	8.91	3.51	0.07	53.39	25.73	13.50	67.00	35.75	0.34	46.16
	**Mean**	**7.94**	**15.50**	**82.35**	**2.14**	**1.09**	**10.44**	**3.46**	**0.07**	**45.17**	**22.37**	**13.38**	**46.90**	**36.59**	**0.38**	**48.53**
	Max	8.70	17.93	87.61	17.72	1.84	16.74	6.34	0.11	68.64	34.46	28.52	75.64	43.49	0.44	64.50
All	Min	7.86	2.66	77.00	0.79	0.72	8.91	3.28	0.07	35.58	17.25	11.90	34.45	34.29	0.08	40.06
	Mean	8.12	8.03	82.13	9.84	1.23	13.19	4.71	0.09	50.60	25.95	19.80	52.51	39.26	0.26	53.62
Background values[a]		—	—	—	—	—	—	—	—	39.78	18.64	13.92	56.61	6.12	0.05	16.85

[a] Data source: Gao, et al. (2007)

Table S7

HMs concentrations in sediments of the Wuliangsu wetland in recent years (mg/kg)

Sampling time	Cr	Ni	Cu	Zn	As	Cd	Pb	Reference
2008	58.90	—	23.66	67.79	11.49	0.17	20.26	Zhao, 2013
2009	36.95	—	19.35	68.79	25.90	0.12	6.6	Zhang et al., 2011
2011	43.7	—	27.9	89.8	12.4	0.19	21.2	Zhao et al., 2013
2010-2016	64.28	—	22.66	68.13	—	—	16.25	Lu et al., 2018
2016	50.60	25.95	19.80	52.51	39.26	0.26	53.62	This study

Table S8

The chemical fraction contents of HMs in the sediments from the Wuliangsuhai wetland

Elements	XDT				NEA				MDP				FCA			
	F1	F2	F3	F4	F1	F2	F3	F4	F1	F2	F3	F4	F1	F2	F3	F4
Cr	1.33	0.52	7.29	90.87	1.23	0.37	7.48	90.91	1.47	0.55	6.32	91.67	1.68	0.94	8.64	88.56
Ni	11.18	4.35	14.95	69.52	10.62	4.68	15.74	68.96	10.16	5.01	16.79	68.04	9.67	3.78	24.80	61.75
Cu	3.30	1.48	41.33	53.88	2.79	4.53	27.01	65.68	4.33	3.75	27.88	64.04	4.33	7.33	27.86	60.48
Zn	14.49	12.20	24.66	48.65	3.30	14.93	29.17	52.60	16.01	14.59	27.86	41.54	22.23	26.91	22.48	28.38
As	1.94	18.59	1.87	77.59	1.07	13.28	0.47	85.17	1.06	20.11	0.74	78.10	1.40	31.99	1.25	65.75
Cd	21.86	33.87	12.66	31.62	25.86	36.18	7.29	30.67	23.32	34.05	12.18	30.46	23.16	35.65	14.60	26.59
Pb	4.79	30.31	33.62	31.27	5.51	28.59	32.40	33.49	5.07	35.74	27.39	31.80	5.60	35.32	21.77	37.31
